# Deterioration of the coercivity due to the diffusion induced interface layer in hard/soft multilayers

**DOI:** 10.1038/srep16212

**Published:** 2015-11-20

**Authors:** Wenjing Si, G. P. Zhao, N. Ran, Y. Peng, F. J. Morvan, X. L. Wan

**Affiliations:** 1 College of Physics and Electronic Engineering, Sichuan Normal University, Chengdu, 610068, China

## Abstract

Hard/soft permanent magnets have aroused many interests in the past two decades because of their potential in achieving giant energy products as well as their rich variety of magnetic behaviors. Nevertheless, the experimental energy products are much smaller than the theoretical ones due to the much smaller coercivity measured in the experiments. In this paper, the deterioration of the coercivity due to the interface atomic diffusion is demonstrated based on a three dimensional (3D) micromagnetic software (OOMMF) and a formula derived for the pinning field in a hard/soft multilayer, which can be applied to both permanent magnets and exchange-coupled-composite (ECC) media. It is found that the formation of the interface layer can decrease the coercivity by roughly 50%, which is responsible for the observed smaller coercivity in both composite and single-phased permanent magnets. A method to enhance the coercivity in these systems is proposed based on the discussions, consistent with recent experiments where excellent magnetic properties are achieved.

Since Kneller and Hawig^1^ proposed the concept of exchange spring in 1991, such a composite material which associates a large coercivity provided by a hard phase and a large remanence offered by a soft phase has been a hot topic in the magnetic academia. The theoretical maximum energy density product, (BH)_max_, as predicted by Skomski and Coey^2^ in 1993, can be as high as 120 MGOe. However, the experimental (BH)_max_ of 20–40 MGOe^3^,^4^,^5^,^6^,^7^,^8^ is much smaller than the predicted one, a phenomenon called “energy product paradox” in some literatures^9^. This paradox is closely related to the coercivity paradox as proposed by Brown^10^,^11^ in 1940 s, where the measured coercivity is much smaller than that predicted by the theory.

A general formula for the pinning field has been obtained for hard/soft multilayers with in-plane crystalline anisotropy:


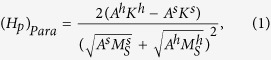


where the superscripts *h* and *s* refer to the hard and soft layers, respectively. A subscript *Para* is used here to denote the case with in-plane crystalline anisotropy. *A*, *K* and *M*_*S*_ represent the exchange energy, the crystalline anisotropy energy and the spontaneous magnetization constants, respectively. This pinning field is derived assuming that the soft layer is thick enough and thus is completely determined by the interface quality, called as self-pinning by Zhao *et al.*^12^,^13^. The self-pinning has been extended to hard/soft composites with other kinds of interfaces (spherical and cylindrical) and to so-called single-phased permanent magnets with soft defects. Such a pinning is found to be the dominant coercivity mechanism in these systems, with the typical pinning field being equal to about 6.7 kOe for Nd_2_Fe_14_B/*α*-Fe systems and 15.4 kOe for SmCo/*α*-Fe systems. These values are close to the measured coercivity in single-phased permanent magnets, but are still much larger than most experimental values in two-phased systems^3^,^4^,^7^,^8^.

The possible atomic diffusion in the interface has been qualitatively discussed^9^,^12^, which can decrease the pinning field by roughly 50%. One can see from Eq. (1) that to retain a large pinning field, the intrinsic magnetic properties of the hard and soft phases need to be highly different. The atomic diffusion can form an interface layer with intrinsic parameters in between those of the soft and hard layers. For a complete magnetic reversal to occur, a domain wall has to overcome two pinning points, from the soft phase to the interface layer and then from the interface layer to the hard phase with the corresponding pinning fields given by:


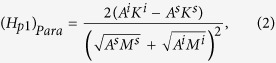



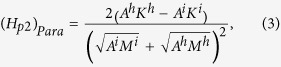


where the superscript *i* stands for the interface layer. Both pinning fields are much smaller than that given by Eq. (1) as the property differences are now reduced considerably.

Similar discussions have been made by Suess *et al.*^14^,^15^ for exchange-coupled-composite (ECC) media^16^ maintaining a good thermal stability at smaller grain sizes. In contrary to permanent magnets, where a large coercivity is preferred, a too high switching field should be avoided for recording media. Therefore, a graded media with a continuous variation in anisotropy along the film normal is proposed^17^,^18^ and experimentally realized^19^ to reduce the property differences between the hard and soft phases, and hence the coercivity. These discussions are mainly based on Eqs (1, 2, 3) given above^14^,^19^.

One has to notice, however, that there is an important difference between the ECC media and the case in deriving Eqs (1, 2, 3). The former usually adopts a perpendicularly magnetic anisotropy (PMA) while an in-plane easy axis has been assumed in deriving Eqs (1, 2, 3). The formulae for perpendicular anisotropy (ECC media) and in-plane anisotropy are exactly the same if the stray field is omitted. However, the shape anisotropy (caused by the stray field) leads to a non-negligible difference between the perpendicular magnetic anisotropy (PMA) case and the in-plane anisotropy case here, namely, contrary to the case with in-plane anisotropy, the stray field for a thin film with out-of-plane anisotropy cannot be ignored. The shape anisotropy for a thin film with perpendicular anisotropy is 2*π*(*M*_*S*_)^2^, which is about 1.03 × 10^7^ erg/cm^3^ for Nd_2_Fe_14_B and comparable to its crystalline anisotropy (4.3 × 10^7^ erg/cm^3^). As shown in Table 1, the equivalent *K*^*h*^ for the out-of-plane case is about 3/4 of that for the in-plane case. More importantly, the equivalent *K*^*s*^ is negative while the absolute value is a few dozen times of that for the in-plane case due to the much larger shape anisotropy (please see Table 1). Thus the spin orientation at the surface of the soft phase obeys the following formula according to the Stoner-Wohlfarth model for a large enough *t*^*s*20^:


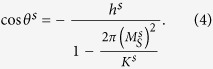


where *h* = *H*/*H*_*K*_ represents the reduced crystalline anisotropy field with *H*_*K*_ = 2*K*/*M*_*S*_. The loop given by Eq. (4) is a non-hysteresis line, which is in sharp contrast with the rectangular hysteresis loop for *θ*^*s*^ with in-plane easy axes, i.e., *θ*^*s*^ is 0° before the magnetization reversal whilst it is 180° after the magnetic reversal according to the SW model.

In this report, the formula of the pinning field for a PMA is derived, which can be applied to both hard/soft permanent magnets and ECC media. The influence of the interface layer due to the atomic diffusion is discussed qualitatively based on this formula and then quantitatively based on a three dimensional (3D) OOMMF calculation.

## Results

Minimizing the total energy density with suitable boundary conditions given in the methods section yields the angular distributions in the soft and hard layers as well as the interface constrains^9^,^20^ for the present calculation. The latter can be written as:


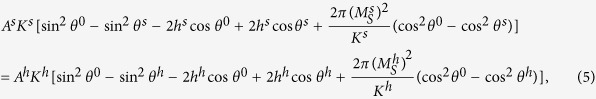



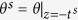
, 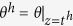 and 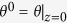 denote the angles of the magnetization at the surfaces of the soft and hard phases as well as the one at the interface. Generally, the exchange coupling of the soft phase is important, which has a significant effect on the reversible demagnetization behavior of the hard phase. However, such an influence can only have an obvious effect on the magnetization of the hard phase within a Bloch wall width^9^,^20^, which is about 4.2 nm for Nd_2_Fe_14_B with an in-plane easy axis (see Table 1).

The magnetizations at further places of the hard phase are not much affected by the exchange coupling due to the existence of the soft phase. Therefore, for a bilayer with *t*^*h*^ larger than one Bloch wall width of the hard layer, *θ*^*h*^ can be calculated^9^,^20^ based on the Stoner-Wohlfarth (SW) model^21^, *i.e. θ*^*h*^ = 0 and *π* before and after the magnetic reversal respectively.

**Figure 1:**
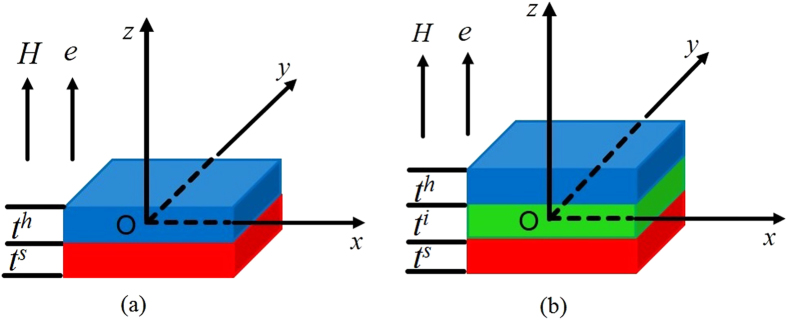
Basic schemes in our work. (**a**), A hard/soft bilayer. (**b**), A hard/interface/soft trilayer.

For a perpendicularly applied field as shown in Fig. 1, the above discussion is also valid, except that the Bloch wall width here is calculated from the effective anisotropy (
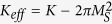
), which is 4.8 nm for Nd_2_Fe_14_B as shown in Table 1.

The approach can be extended to the soft phase with the effective easy axis perpendicular to the applied field, where two situations can occur. In the first situation *θ*^*s*^ can reach 180° before the depinning takes place so that the pinning field can be obtained by replacing the anisotropies in Eq. (1) with *K*_*eff*_ = *K* − 2π(*M*_*S*_)^2^ , which is:


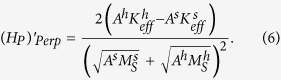


The prerequisite for the above equation to be valid is that the derived pinning field is larger than (*H*_*K*_)_*eff*_ so that Eq. (4) has no solution indicating that *θ*^*s*^ reached 180° before the depinning takes place. Comparing 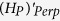  in Table 2 with (*H*_*K*_)_*eff*_ in Table 1 shows that this situation cannot occur for all hard/soft multilayers. In particular, for Nd_2_Fe_14_B/Fe bilayers, 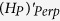 = 14.9 kOe is much smaller than the corresponding (*H*_*K*_)_*eff*_ of 51.1 kOe.

In the other situation *θ*^*s*^ is smaller than 180° at the pinning state with the exact value given by Eq. (4), which is about 120° for a Nd_2_Fe_14_B/*α*-Fe bilayer as shown in Supplementary Fig. S1 in the supplementary information. Combining Eqs (4) and (5) with *θ*^*h*^ = 0 considered, we arrive at an important analytical formula for the pinning field :


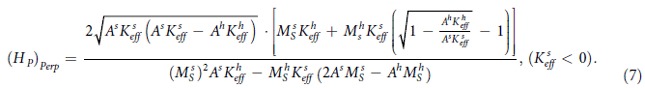


Such an explicit formula, although is much more complicated than Eq. (1) for the case with the in-plane anisotropy, can be used in a wider thickness region for the soft phase. Both Eqs (7) and (1) are derived in the limit of infinite thick layers so that the spin orientations in the surface of the layers obey the SW model. However, “infinite” here is relative in comparison with the corresponding effective exchange length. Please note that it is the effective exchange length rather than the normal exchange length of the soft phase which decides the criterion. The effective exchange length of the soft phase is much reduced for the PMA case due to its large shape anisotropy in comparison with the crystalline anisotropy and thus makes the difference.

In more detail, Eq. (1) is used in the case with an in-plane crystalline anisotropy, where the thickness of the soft layer is larger or in comparison with the corresponding exchange length *l*_*ex*_ = (*A*/*K*)^1/2^ = *Δ*/*π*. The latter is usually very large (see Table 1) so that Eq. (1) can be used only when t^s^ is larger than 15 nm for Nd_2_Fe_14_B/*α*-Fe bilayer systems. In this case, the composite system is in decoupled region, where the nucleation field and energy product are very small^9^,^22^.

For a system with a PMA discussed in the present work, the absolute value of the effective anisotropy for the soft phase is much increased with the shape anisotropy (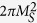) taken into account. Thus the corresponding effective domain wall width in the thickness direction and the corresponding exchange length reduce significantly, as shown in Table 1. In this case, the soft layer thickness, which is larger than 1/3 of its effective domain wall width (corresponding to about 60° for a 180° domain wall), can be taken as “infinite”. The criterion here for PMA is much smaller than that for in-plane anisotropy. This estimation is also consistent with the more rigorous (but much more complicated) 1D calculations given in ref. [23] for a hard/soft multilayer with in-plane anisotropy and [20] for a multilayer with PMA respectively. As shown in Supplementary Fig. S2 (a), the calculated pinning field is about a constant when *t*^*s*^ is larger than about 5 nm for a system with PMA. In comparison, as shown in Supplementary Fig. S2 (b), the pinning field reaches a constant only when *t*^*s*^ is larger than about 15 nm for in-plane anisotropy case. The calculated pinning fields based on the more rigorous 1D model and the relative error in using Eqs (7) and (1) are summarized in Supplementary Table S1 to justify this issue. As a result, Eq. (7) can be used when t^s^ is larger than 5 nm for Nd_2_Fe_14_B/*α*-Fe bilayer systems with a PMA (see Table 1), a condition normally satisfied for an optimized composite layered system.

In order to understand the magnetic behavior of the interface layer quantitatively, a 3D software (OOMMF) has to be used to calculate the hysteresis loops and magnetic reversal processes of Nd_2_Fe_14_B/α-Fe systems. In the present work, we adopt a square layered system similar to those used in refs. [23,^24^] for Nd_2_Fe_14_B/*α*-Fe systems, as shown in Fig. 1. The magnetocrystalline axes of all the layers and the applied field are assumed to be oriented along the *z* direction.

**Figure 2:**
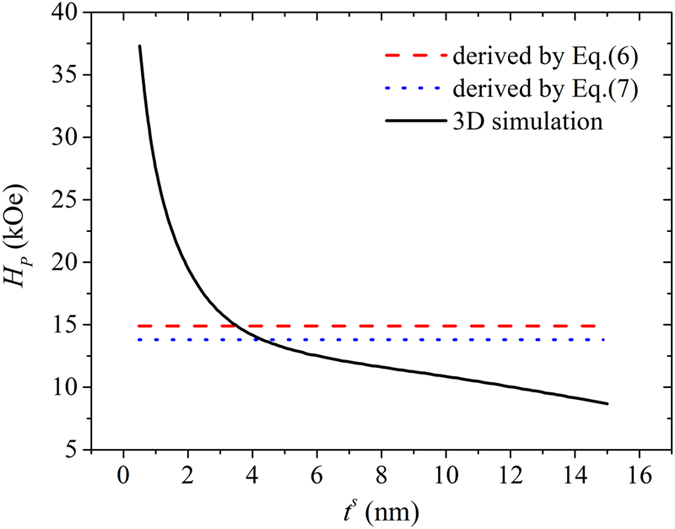
Fe_14_B(10 nm)/*α*-Fe(*t*^*s*^ nm) according to Eqs (6) and (7) along with those based on the OOMMF simulations where the easy axes is perpendicular to the film plane.

The parameters adopted for the bilayers in this work are shown in Table 1, with the free boundary conditions applied. Figure 2 compares the calculated pinning fields for Nd_2_Fe_14_B/*α*-Fe bilayers under various soft layer thicknesses *t*^*s*^ according to Eqs (6) and (7) as well as those based on the OOMMF simulations. One can see that the pinning field according to Eq. (6) is larger than that based on Eq. (7), indicating that the situation where *θ*^*s*^ smaller than 180° at the pinning state is more energy favorable with less pinning.

On the other hand, the parameters of the interface layer are believed to be in close relation with those of the hard and soft layers, which is assumed to be a linear interpolation of those for the main phases in this work:


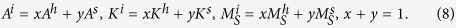


For the magnetization to reverse smoothly, the two pinning fields *Hp1* and *Hp2* are assumed to be equal in this calculation, which represent the pinning field between the hard and interface layers and that between the interface and soft layers respectively:


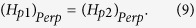


For convenience, this pinning field is denoted as 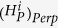 in the following. For multilayers with in-plane anisotropy, the effective anisotropies of both hard and soft phases are positive^9^,^12^, so that the interface layer also has a positive effective anisotropy which is in between those of the soft and hard layers. Therefore, the two pinning fields can be given by Eqs (2) and (3) based on Eq. (1). In the present calculation, positive and negative *K*_*eff*_ are obtained for the hard and soft layers respectively whilst a positive *K*_*eff*_ is adopted for the interface layer as listed in Table 3 since 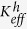 dominates over 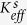. Therefore the pinning field between the hard and interface layers and that between the interface and soft layers are based on Eqs (1) and (7) respectively, which are given in Table 2 for various materials.

From Eqs (8) and (9), the parameters for the interface layers can be determined, as listed in Table 3.

**Figure 3:**
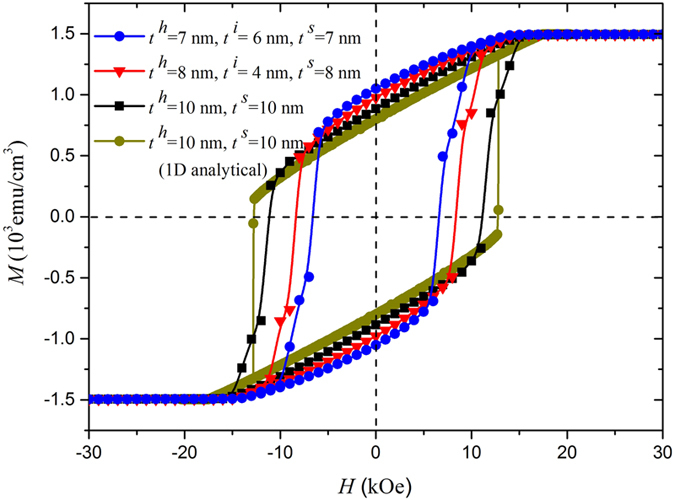
Comparison of calculated hysteresis loops for Nd_2_Fe_14_B(10 nm)/*α*-Fe(10 nm) and Nd_2_Fe_14_B(*t*^*h*^)/interlayer(*t*^*i*^)/*α*-Fe(*t*^*s*^) with easy axes perpendicular to the film plane (corresponding to Fig. 1(a,b) respectively). The blue stars stand for *t*^*h*^ = *t*^*s*^ = 7 nm and *t*^*i*^ = 6 nm, the red triangles for *t*^*h*^ = *t*^*s*^ = 8 nm and *t*^*i*^ = 4 nm, the black squares and the green circles for *t*^*h*^ = *t*^*s*^ = 10 nm and *t*^*i*^ = 0 nm, using OOMMF and 1D analytical respectively.

Hysteresis loops and spatial spin distributions^23^,^24^ have been calculated for various hard/soft multilayers based on OOMMF software. The spin distributions are generally similar to those without the interface layer, as have been reported in refs. 23 and 24. Figure 3 shows the hysteresis loops of the Nd_2_Fe_14_B(10 nm)/*α*-Fe(10 nm) system with various thicknesses of interface layer. The loops based on OOMMF and one dimensional (1D) analytical calculation for the bilayer without the interface layer agree well with each other, justifying our calculation. The way to calculate the 1D hysteresis loops has been given in the methods. The calculated coercivity is reduced considerably when an interface layer is formed between the hard and soft phases whereas both the remanence and the nucleation field do not vary much. Coercivities for the systems with 4-nm-thick and 6-nm-thick interface layers decrease to 8.4 kOe and 6.5 kOe respectively. Further increase of *t*^*i*^ might reduce the coercivity by more than 50% in comparison with the case without the interface layer, as can be estimated from Eqs (1) and (7) and shown in Table 2. As a matter of fact, the difference of domain wall energy between the hard and the soft layer 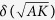 is larger in systems without interlayer than in systems that have formed one, the latter situation resulting in a smaller coercivity. This finding may explain the coercivity paradox^10^ in both single-phased and composite permanent magnets in some degree. In particular, similar to the multilayer systems, an interface layer can be formed between the soft defects and the main phases in the so-called single-phased permanent magnets, which decreases the coercivity significantly.

## Discussion

**Figure 4:**
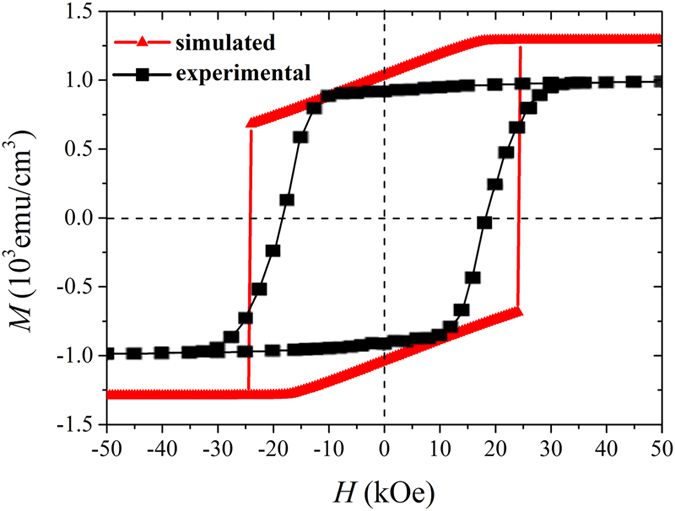
Comparison of calculated and experimental hysteresis loops for [Nd_2_Fe_14_B(30 nm)/Nd(3 nm)/Ta(1 nm)/Fe_2_Co(10 nm)/Ta(1 nm)]_4_/Nd_2_Fe_14_B(30 nm)/Nd(3 nm) multilayers.

These results imply a possible way to enhance the coercivity in hard/soft multilayers: inserting a non-magnetic layer in between the hard and soft phases to inhibit the atomic diffusion near the interface. Experimentally, Cui *et al.*^25^ inserted a Ta spacer layer in Nd_2_Fe_14_B and FeCo nanocomposite films to achieve a high coercivity and hence a high (BH)_max_ of 486 KJ/m^3^. Figure 4 compares the calculated hysteresis loop based on OOMMF and the experimental data obtained by Cui *et al.*^25^. One can see that the two loops are consistent with each other, justifying our theory. In particular, the calculated coercivity is about 24 kOe, which is only slightly larger than the experimental data (about 18 kOe), demonstrating a significant enhancement of coercivity in comparison with Fig. 3 where an interface layer is formed. Therefore, Brown’s coercivity paradox can be overcome in these systems in principle, along with the associated energy product paradox.

The method can be extended to other hard/soft composite systems. Li *et al.*^26^ used the severe plastic deformation (SPD) method to inhibit the formation of the metastable intermediate phases at the Nd_2_Fe_14_B/*a*-Fe interface in bulk nanocomposite magnets. The coercivity is thus enhanced from 4.5 kOe to 7.0 kOe, consistent with those shown in Table 2.

The above discussions can be applied to the ECC as well as the graded media. For example, the pinning field for a FePt/*α*-Fe bilayer with PMA is about 18 kOe, which reduces to 7 kOe when an interface layer is formed (see Table 2). The latter agrees well with the experimental coercivity of 8 kOe for a FePt/*α*-Fe bilayer given in ref. 19. As for the graded media, the estimated pinning fields due to the variation of crystalline anisotropy^19^ based on Eqs (1) and (7) ranges from 1.6 kOe to 3.5 kOe. Such a large variation of the switching fields can explain the significant slant in the loop observed for the graded media, where the experimental coercivity is about 2.2 kOe.

In summary, a formula for the pinning field in a hard/soft multilayer with PMA has been derived using 1D micromagnetics, which can be used in a wide thickness region. The coercivity is much reduced because of the possible interface atomic diffusion according to the 3D micromagnetics, consistent with the 1D predictions. The formula and results can be applied to both permanent magnets and exchange-coupled-composite (ECC) media. The coercivity might drop by 50% due to the formation of the interface layer, responsible for the much smaller coercivity observed in both composite and single-phased permanent magnets. Finally we propose a method to enhance the coercivity in hard/soft composite systems, justified by recent experiments in both multilayers and bulk nanocomposite magnets.

## Methods

The 1D calculations start from the total energy density of a hard/soft bilayer system with PMA^20^,^27^:


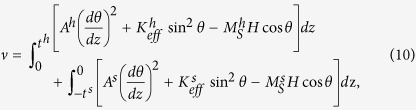


where *θ* is the angle between the magnetization vector and the applied field, whilst *K*_*eff*_ = *K* - 2*π*(*M*_*S*_)^2^ denotes the effective anisotropy and 2*π*(*M*_*S*_)^2^ is the shape anisotropy. Eq. (10) includes the energy of both soft and hard phases, where three energy terms have been considered in each phase, *i.e.*, exchange energy, effective anisotropy energy and Zeeman energy as shown in the bracket of Eq. (10). Such an expression of the total energy is the same as that for a hard/soft system with an in-plane easy axis^9^,^22^. An important difference is that 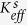 here is negative whereas it is a small positive value in the latter case. The energy for a hard/soft multilayer system is similar, with the soft layer thickness *t*^*s*^ and the hard layer thickness *t*^*h*^ replaced by *t*^*s*^/2 and *t*^*h*^/2 respectively^27^,^28^. The calculated hysteresis loops and spin distributions also have such a correspondence.

Substituting Eq. (10) into Euler - Lagrange equation 
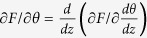
, which is obtained through the variation of the volume part of the total energy, we arrive at the implicit formulas for the magnetization orientation *θ* at the thickness direction:


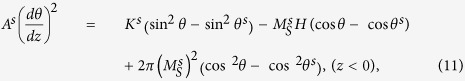



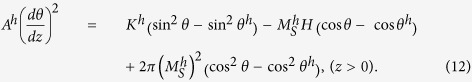


Integration of Eqs (11) and (12) yields the relation between *θ* and *z:*


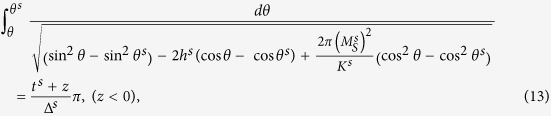



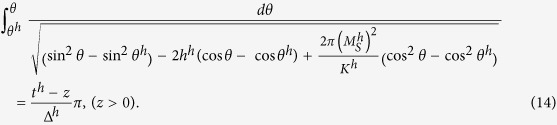


The specific angles are defined as following:


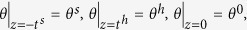


which denote the orientation of the magnetization at the hard and soft surfaces as well as that at the interface. In addition, the interface restrain can be obtained through the variation of the surface contributions to the total energy, which is the Carl-Weierstrass relation:


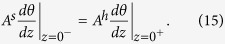


Similar restraints can be obtained at hard and soft surfaces:


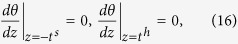


as the d*θ*/dz in nonmagnetic phase can be regarded as 0. Eq. (16) serve as the boundary conditions^9^,^12^,^13^ for the present calculation. Further, substituting Eqs (11) and (12) into Eq. (15), we arrive at Eq. (5) given in the results. Eqs (5), (13) and (14) are the basic equations to calculate the spin orientations and hysteresis loops, which have to be solved with the aid of the numerical method as both Eqs (13) and (14) are elliptical integrations. Replacing *θ* by *θ*^*0*^ in the limits of integration for Eqs (13) and (14) yields three equations, *i.e*., Eq. (5) and updated Eqs (13) and (14), from which *θ*^*s*^, *θ*^*0*^ and *θ*^*h*^can be obtained. Now we can solve Eqs (13) and (14) to find *θ* for different z, the spin distributions and hence the hysteresis loops.

The 3D micromagnetic calculation of the software OOMMF is based on a Landau-Lifshitz-Gilbert dynamic equation^29^:


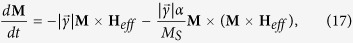


where **M** is the magnetization, **H**_*eff*_is the effective field, 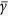 is the Landau-Lifshitz gyromagnetic ratio, and *α* is a dimensionless damping constant. The effective field is defined as follows:


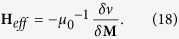


The average energy density *ν* is a function of **M** specified by Brown’s equations^2^,^10^:


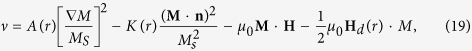


where **H** and **H**_*d*_(r) are the applied and magnetostatic self-interaction fields while *M*_*S*_* = M(r)* is the spontaneous magnetization. These equations hold for all layers. The four terms at the right side of Eq. (19) correspond to the exchange, anisotropy, applied field (Zeeman) and magnetostatic (demagnetization) energies respectively.

The length and width of all layers are set as 300 nm for the calculation so that a film shape is kept, including both hard and soft phases as well as the interface layer. The length and width of each cell are 3 nm, and the height of each cell is set as 1 nm. The applied field varies from 60 kOe to −60 kOe, starting from a positive saturation state, where the initial magnetization is parallel to the applied field. The exchange interaction between the neighboring atomic pair is taken into account and the exchange coupling at the interface is set as 1 × 10^−6^ erg/cm for Nd_2_Fe_14_B/*α*-Fe systems.

## Additional Information

**How to cite this article**: Si, W. *et al.* Deterioration of the coercivity due to the diffusion induced interface layer in hard/soft multilayers. *Sci. Rep.* **5**, 16212; doi: 10.1038/srep16212 (2015).

## Supplementary Material

Supplementary Information

## Figures and Tables

**Table 1:** Intrinsic magnetic properties for various magnetic materials.

Material	*A*(erg/cm)	*K*(erg/cm^3^)	*M*_*S*_(emu/cm^3^)	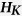 (kOe)	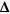 _(nm)_	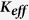 (erg/cm^3^)	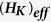 (kOe)	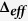 (nm)	Refs.
Nd_2_Fe_14_B	7.70 × 10^−7^	4.30 × 10^7^	1.28 × 10^3^	67.19	4.20	3.27 × 10^7^	51.1	4.82	9,30
*α*-Fe	2.50 × 10^−6^	4.60 × 10^5^	1.71 × 10^3^	0.54	73.20	−1.79 × 10^7^	−21.0	11.74	9,30
FePt	1.25 × 10^−6^	2.50 × 10^7^	5.00 × 10^2^	100.00	7.02	2.34 × 10^7^	93.6	7.26	31
SmCo	1.20 × 10^−6^	5.00 × 10^7^	5.50 × 10^2^	181.82	4.87	3.81 × 10^7^	138.5	0.56	32,33
Fe_2_Co_1_	1.67 × 10^−6^	1.00 × 10^2^	1.87 × 10^3^	1.07 × 10^−4^	4.06 × 10^3^	−2.20 × 10^7^	−23.5	8.66	2,25

*A*, *K*, *M*_*S*_, *H*_*K*_and *Δ* denote the exchange constant, the crystalline anisotropy constant, the spontaneous magnetization, the anisotropy field and the Bloch wall width respectively. *K*_*eff*_, (*H*_*K*_)_*eff*_ and *Δ*_*eff*_are the corresponding effective constants with the shape anisotropy taken into account.

**Table 2:** Calculated pinning fields for bilayers without interface layer and those with interface layers.

Hard/soft systems	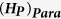 (kOe)	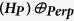 (kOe)	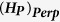 (kOe)	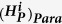 (kOe)	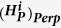 (kOe)
Nd_2_Fe_14_B/*α*-Fe	6.8	14.9	13.8	2.76	4.12
FePt/*α*-Fe	7.4	18.1	17.8	3.36	6.99
SmCo/*α*-Fe	11.3	21.8	18.7	6.39	10.87
Nd_2_Fe_14_B/Fe_2_Co_1_	8.7	16.3	14.8	3.94	5.29

(*H*_*p*_)_*para*_, 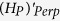 and 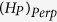 are the pinning fields for the bilayers with in-plane and PMA based on Eq. (1), (6) and (7) respectively, whilst 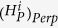 denotes the pinning field between the hard/soft layer and the interface layer with parameters given in Table 3. 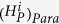 is the corresponding pinning field with the in-plane easy axes.

**Table 3:** Adopted magnetic properties of interface layer for various hard/soft bilayers with the crystalline easy axes perpendicular to the film plane based on Eqs (8) and (9), where the superscript *i* stands for the interface layer.

Interface	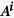 (erg/cm)	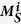 (emu/cm^3^)	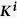 (erg/cm^3^)	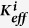 (erg/cm^3^)	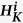 (kOe)	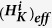 (kOe)	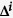 _(nm)_	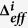 (nm)
Nd_2_Fe_14_B/*α*-Fe	1.65 × 10^−6^	1.50 × 10^3^	2.12 × 10^7^	7.08 × 10^6^	28.3	9.4	8.76	15.17
FePt/*α-*Fe	1.70 × 10^−6^	9.34 × 10^2^	1.41 × 10^7^	8.59 × 10^6^	30.2	18.4	10.91	13.98
SmCo/*α*-Fe	1.81 × 10^−6^	1.09 × 10^3^	2.47 × 10^7^	1.72 × 10^7^	45.3	31.6	8.50	10.19
Nd_2_Fe_14_B/Fe_2_Co_1_	1.15 × 10^−6^	1.53 × 10^3^	2.42 × 10^7^	9.48 × 10^6^	31.6	12.4	6.85	10.94
