# Supplementary material for: Deterioration of the coercivity due to the diffusion induced interface layer in hard/soft multilayers
Source: Sci Rep. 2015 Nov 20;5:16212. doi: 10.1038/srep16212 (PMC4653640; doi:10.1038/srep16212)
Supplement: Supplementary Information [file srep16212-s1.pdf]

Deterioration of the coercivity due to the diffusion induced interface layer in hard/soft multilayers

WenJing Si[1], G. P. Zhao[1,*], N. Ran[1], Y. Peng[1], F. J. Morvan[1], X. L. Wan[1]

[1]*College of Physics and Electronic Engineering, Sichuan Normal University, Chengdu, 610068, China*

*correspondence to zhaogp@uestc.edu.cn

SUPPLEMENTARY INFORMATION

Supplementary Note 1: Angular distributions of magnetization in the thickness direction based on OOMMF[1] calculations.

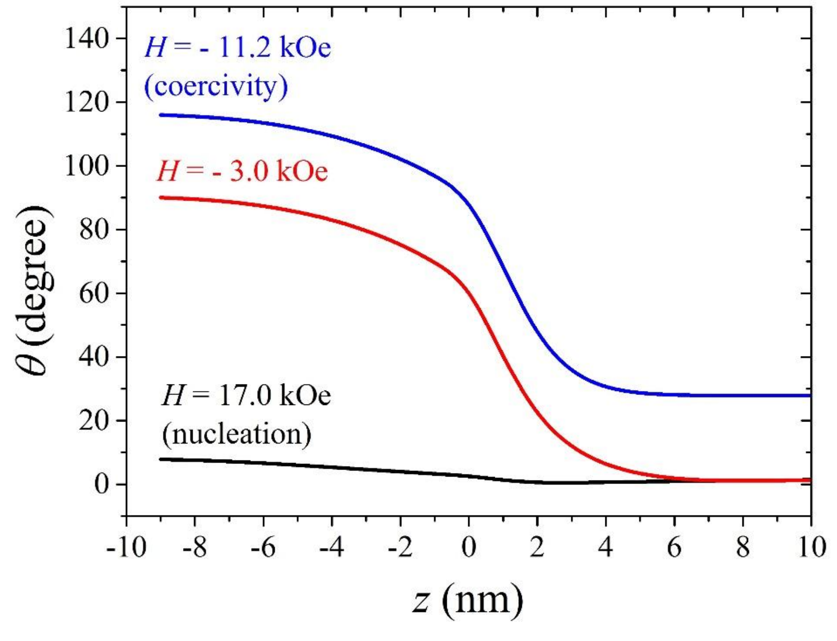

Fig. S1. Angular distributions of the magnetization in the thickness direction for $Nd_2Fe_{14}B$(10 nm)/α-Fe(10 nm) bilayer under various applied fields H.

Angular distributions of the magnetic moments in the thickness direction have been calculated by OOMMF for $Nd_2Fe_{14}B$(10 nm)/α-Fe(10 nm) bilayer without any interface layer, which are shown in Fig. S1 for three different applied fields. Nucleation of a prototype of domain wall takes place at a positive applied field H = 17 kOe whilst

the domain wall depinning occurs at the H = -11.2 kOe. At the coercive point (H = -11.2 kOe), the whole system holds a domain wall less than 120° in the thickness direction, where the hard and soft layers each holds half. Similar angular distributions can be found for other values of the soft layer thickness, as shown in Figs. 4 and 5 of Ref. [2].

Supplementary Note 2: The pinning fields based on a more rigorous 1D analytical calculation given by Refs. [3] and [4].

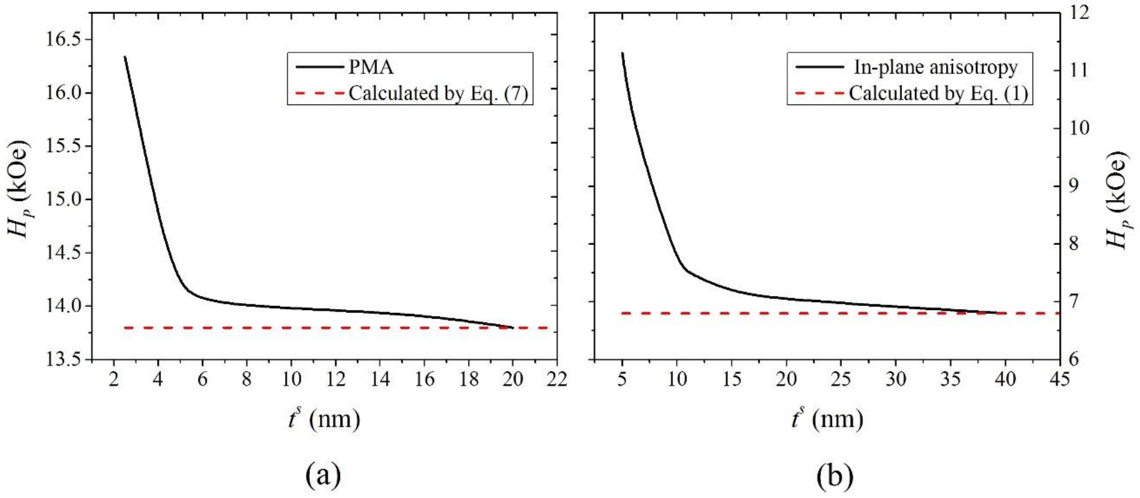

Fig. S2. Pinning fields for $Nd_2Fe_{14}B$ (10 nm)/α-Fe (various thickness) bilayer with (a) a PMA and (b) in-plane anisotropy based on a more rigorous 1D analytical calculation given by Refs. [3] and [4]. The pinning fields based on Eqs. (1) and (7) are shown for comparison.

As t^s increases, the pinning field in the left figure decreases rapidly and reaches a constant when $t^s \geq 5$ nm. The pinning field for a system with in-plane anisotropy is only about half of that with a PMA for large t^s, which drops more gradually in comparison as t^s increases and reaches a constant only when $t^s \geq 15$ nm. The dashed lines show the calculated pinning fields based on Eqs. (1) and (7) in the present paper respectively.

Supplementary Note 3: Table of the relative errors for the pinning fields based on Eq. (1) and (7).

	In-plane anisotropy $(H_P)_{Para}$ = 6.8 kOe					PMA $(H_P)_{Perp}$ = 13.8 kOe				
t^s (nm)	5	10	15	20	40	2.5	4	5	15	20
H_P (kOe)	11.3	7.67	7.16	7.03	6.8	16.34	14.83	14.2	13.95	13.8
Relative error (%)	39.8%	11.3%	5.0%	3.3%	0	15.5%	6.9%	2.8%	1.1%	0

Table S1. | Comparison of the pinning fields based on Eq. (1) and (7) and those by a more rigorous 1D analytical calculation given by Refs. [3] and [4] with the relative errors listed.

The relative error of Eq. (7), given by $\frac{\left|(H_p)_{Perp} - H_p\right|}{H_p}$, is smaller than 3% for $t^s > 5$ nm On the other hand, the relative error of Eq. (1), given by $\frac{\left|(H_p)_{Para} - H_p\right|}{H_p}$, is smaller than 5% only for $t^s > 15$ nm.

Supplementary Note 4: The shape anisotropy energy calculation based on the 1D model and on the average magnetization vector, along with the demagnetization energy calculation based on the 3D software OOMMF.

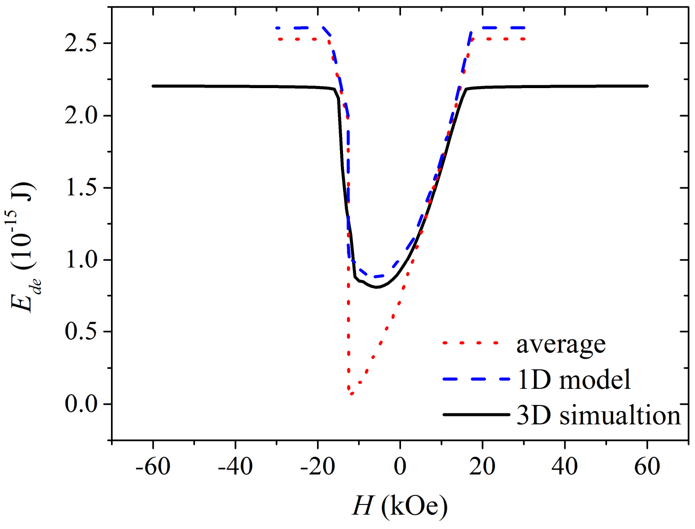

Fig. S3. Comparison of the calculated shape anisotropy energy based on the 1D model (blue dashed) and the demagnetization energy based on the 3D OOMMF calculation for $Nd_2Fe_{14}B$(10 nm)/α-Fe(10 nm) bilayer. The estimated shape anisotropy energy based on the average magnetization vector (red dot) is also shown for comparison.

One can see that the simplification adopted in the present 1D calculation (blue dashed) is generally good in comparison with the OOMMF results. In particular, at the coercive point (H = -12.6 kOe) the total magnetization is 0 and hence the shape anisotropy is significantly reduced, agreeing well with the OOMMF calculation. In this state, the soft layer points down and the hard layer up. However, it should also be noticed that even in this case the stray field energy is far away from 0, roughly half of that for the saturation state according to the OOMMF calculation. The largest error occurs at the saturation state where our 1D method overestimates the demagnetization energy by 17%. The other possible approach to estimate the shape anisotropy within the 1D model is to consider the average magnetization vector, with the shape anisotropy energy density given by $2\pi\left[\overline{M_S(z)\cos\theta}\right]^2$, which can reduce the error at the saturation state by roughly 2%. However, for the nonuniform case this simplification causes larger errors. In particular, at the coercive point, which is the focus of the present work, the vector average of the total magnetization is zero so that such a method underestimates the stray field energy considerably.

References in Supplementary

1. Donahue, M. J. & Porter, D. G. *OOMMF User's guide, Version 1.0, Interagency Report NISTIR 6376* (Gaithersburg, MD, 1999).

2. Yuan, X. H., *et al.* 3D and 1D calculation of hysteresis loops and energy products for anisotropic nanocomposite films with perpendicular anisotropy. *J. Magn. Magn. Mater.* **343**, 245-250 (2013).

3. Zhao, G. P. & Wang, X. L. Nucleation, pinning, and coercivity in magnetic

nanosystems: An analytical micromagnetic approach. *Phys. Rev. B* **74**, 012409 (2006).

4. Zhao, G. P., Bo, N., Zhang, H. W., Feng, Y. P. & Deng, Y. Demagnetization process and hysteresis loops in perpendicularly oriented hard/soft trilayers. *J. Appl. Phys.* **107**, 083907 (2010).
